# Solving the Chinese Finger Trap: A Novel Method for Simultaneous Superior and Inferior Vena Cava Filter Removal

**DOI:** 10.7759/cureus.3400

**Published:** 2018-10-02

**Authors:** Taylor S Harmon, Mario Agrait, Brian Sifrig, Jerry Matteo, Travis E Meyer

**Affiliations:** 1 Radiology, The University of Texas Medical Branch, Galveston, USA; 2 Radiology, University of Florida College of Medicine, Jacksonville, USA

**Keywords:** svc filter, ivc filter, retrieval, interventional radiology, anticoagulation, deep venous thrombosis, internal jugular vein, venogram, fragmentation, snare

## Abstract

As many as 130,000 inferior vena cava (IVC) filters are placed annually, with as few as 5,000 retrieved a year for patients who no longer require protection from deep vein thrombosis. Superior vena cava (SVC) filter placement is an even less common occurrence and is usually reserved for special cases. Furthermore, the simultaneous placement of IVC and SVC filters is most rare, whereas simultaneous IVC and SVC filter removal has not been reported in the literature. We present a case and a novel technique for successful concurrent removal of IVC and SVC filters in a patient.

## Introduction

The long term risks associated with inferior vena cava (IVC) filters are well documented and include, but are not limited to, lower extremity deep vein thrombosis (DVT), filter fracture, filter migration, filter embolization, and IVC perforation [1]. These risks are always conveyed to the patient before the patient receives a filter placement, in addition to disclosing the possibility of permanently leaving the filter in the IVC. Typically, IVC filters are removed once the patient is no longer susceptible to various forms of coagulopathy and is an adequate candidate for elective filter retrieval. In that case, there are methods for retrieval that are colloquially accepted amongst interventionists [2].

When reviewing the literature for risks and complications of superior vena cava (SVC) filter placement, we found that data is limited, though existent [3]. The complications are similar to that of IVC filter placement; however, placement of SVC filters is controversial and dependent upon specific patient presentations [4]. Furthermore, though there have been documented instances of SVC filter placement in some patients, SVC filter removal has hardly ever been reported [5]. After an extensive review to identify possible complications of concurrent SVC/IVC filter removal, we found that the literature does not recognize such cases or concomitant risks. The following case will elucidate a novel and appropriate method for removing both SVC and IVC filters concurrently in a patient who desired to do so and was an appropriate elective candidate.

## Technical report

This is a case of a 72-year-old African-American male who was diagnosed with a 9 cm right frontotemporal meningioma and who underwent tumor embolization followed by craniotomy and resection. After successful excision of the parenchymal mass, the patient underwent placement of a retrievable IVC filter by vascular surgery for lower extremity venous thromboembolism prophylaxis. However, the patient developed bilateral upper extremity swelling and was diagnosed with bilateral upper extremity deep venous thrombosis (DVTs). Due to the patient’s recent neurosurgical intervention, the risk of hemorrhage from oral anticoagulation was too great. As a result, interventional radiology was consulted for the placement of an SVC filter. After discussion with the patient, the decision was made to place an additional retrievable filter in the SVC, with the intention of having both IVC and SVC filters removed in the future.

After six months with no evidence of DVT formation on bilateral upper and lower extremity ultrasound examinations, the patient was placed on oral anticoagulation. At this time, the patient continued to desire to have both SVC and IVC filters removed. The following discloses the novel simultaneous retrieval of both SVC and IVC filters.

Step 1. Gaining dual access to the right internal jugular and femoral veins

Using ultrasound guidance, a micropuncture kit was used to gain access to the right internal jugular vein (IJV) and, subsequently, the right femoral vein. A 180 cm Amplatz Guidewire (Boston Scientific, Marlborough, MA) was carefully placed in the IVC, as to avoid ensnaring either SVC or IVC filters. Subsequently, the same guidewire was introduced through the femoral vein into the SVC in a similar manner.

**Figure 1:**
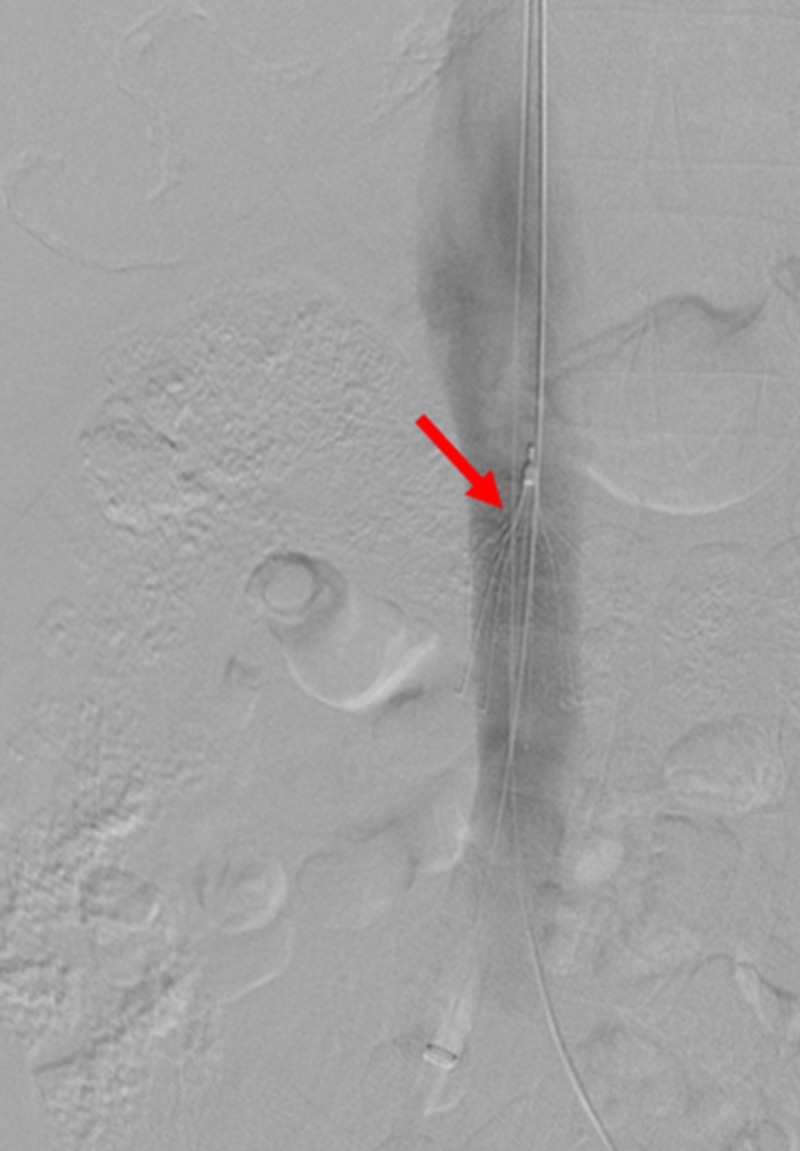
Initial Venogram of the Inferior Vena Cava A venogram is shown of the inferior vena cava and inferior vena cava filter (red arrow) with no intraluminal clot.

Over the Amplatz Guidewire, a 15 French 33 cm DrySeal Flex Sheath (W. L. Gore & Associates, Inc., Newark, DE) was placed into the IVC below the level of the IVC filter from the femoral access. A venogram was performed demonstrating the retrievable Denali Vena Cava Filter (Bard Peripheral Vascular, Inc., Tempe, Arizona) with no evidence of intraluminal clot and only mild tilting (Figure 1).

**Figure 2:**
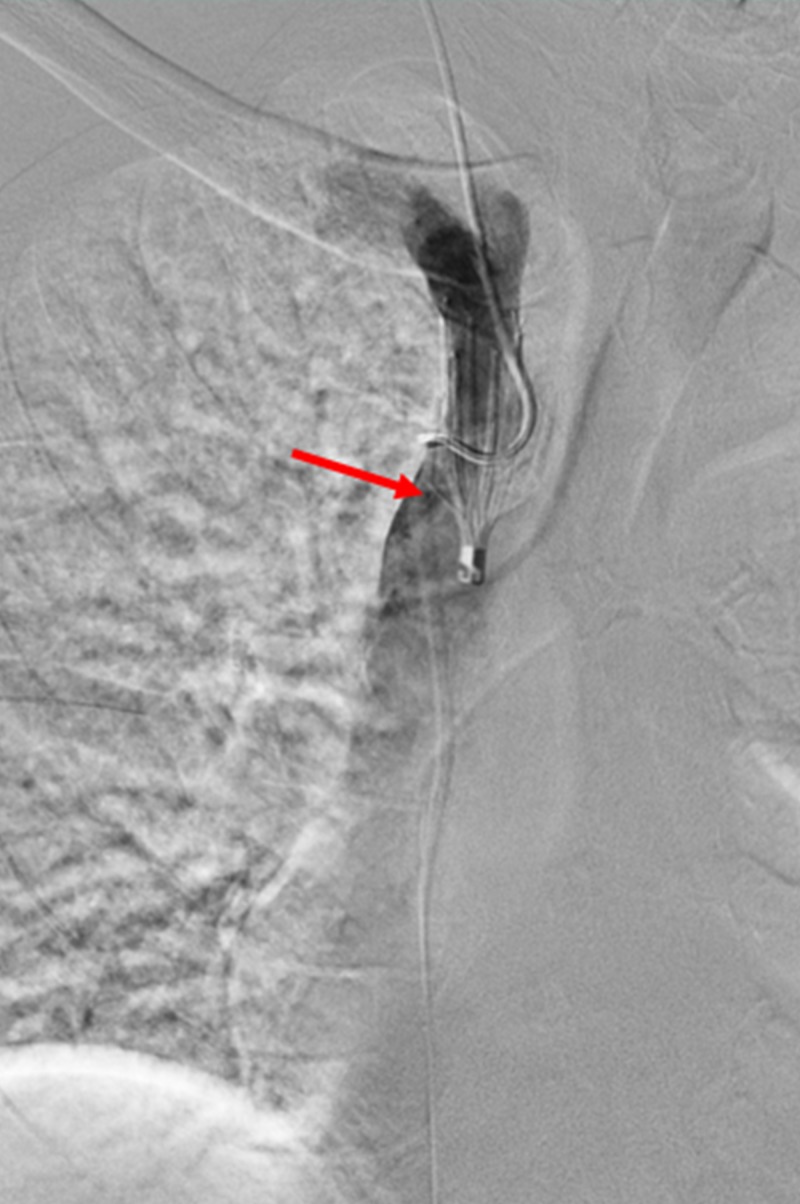
Initial Subtracted Venogram of the Superior Vena Cava A subtracted venogram of the superior vena cava filter (red arrow) that shows tilting towards the brachiocephalic vein with an unembedded tip.

Over the right IJV guidewire, a Contra Flush Catheter (Boston Scientific, Marlborough, MA) was introduced into the SVC. A venogram was performed and showed a retrievable Denali Vena Cava Filter (Bard Peripheral Vascular, Inc., Tempe, Arizona), demonstrating moderate medial tilting towards the brachiocephalic vein. There appeared to be no evidence of SVC abnormality or intraluminal clot (Figure 2).

Step 2. Advancement through the IVC filter with the protective stabilizing sheath

**Figure 3:**
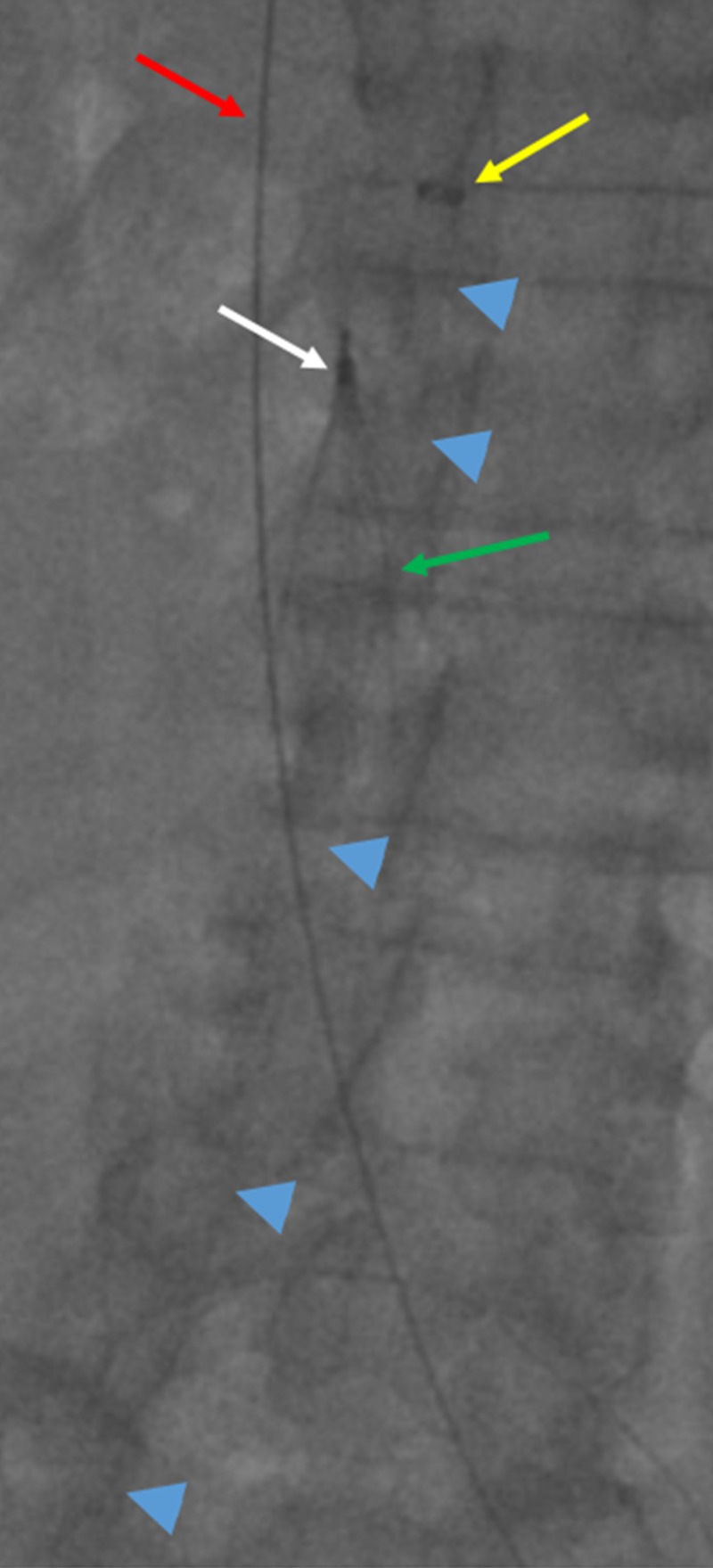
Stabilizing Sheath Placement Through the Inferior Vena Cava Filter A low dose cropped fluoroscopy image of the abdomen shows a retrievable inferior vena cava filter at the level of the second lumbar vertebral body (white arrow). The 15 French DrySeal Flex Sheath is outlined with the blue arrow heads, and the distal end is superior to the inferior vena cava filter (yellow arrow). This sheath originates from a right groin approach and passes through the filter (green arrow). A guidewire from the internal jugular approach is seen passing through the inferior vena cava filter as well (red arrow).

A 15 French DrySeal Flex Sheath was carefully guided through the IVC filter struts with the introducer in place. The introducer was removed once the sheath was in the proximal IVC (Figure 3).

An 8 French Pinnacle Destination Guiding Sheath (Terumo Medical, Somerset, NJ) was advanced through the larger sheath and placed close to the tip of the SVC filter. The protection of the 15 French sheath allowed for minimal disruption of the IVC filter while passing a smaller 8 French sheath through it, in an attempt to access the SVC filter.

Step 3: Removal of the SVC filter

**Figure 4:**
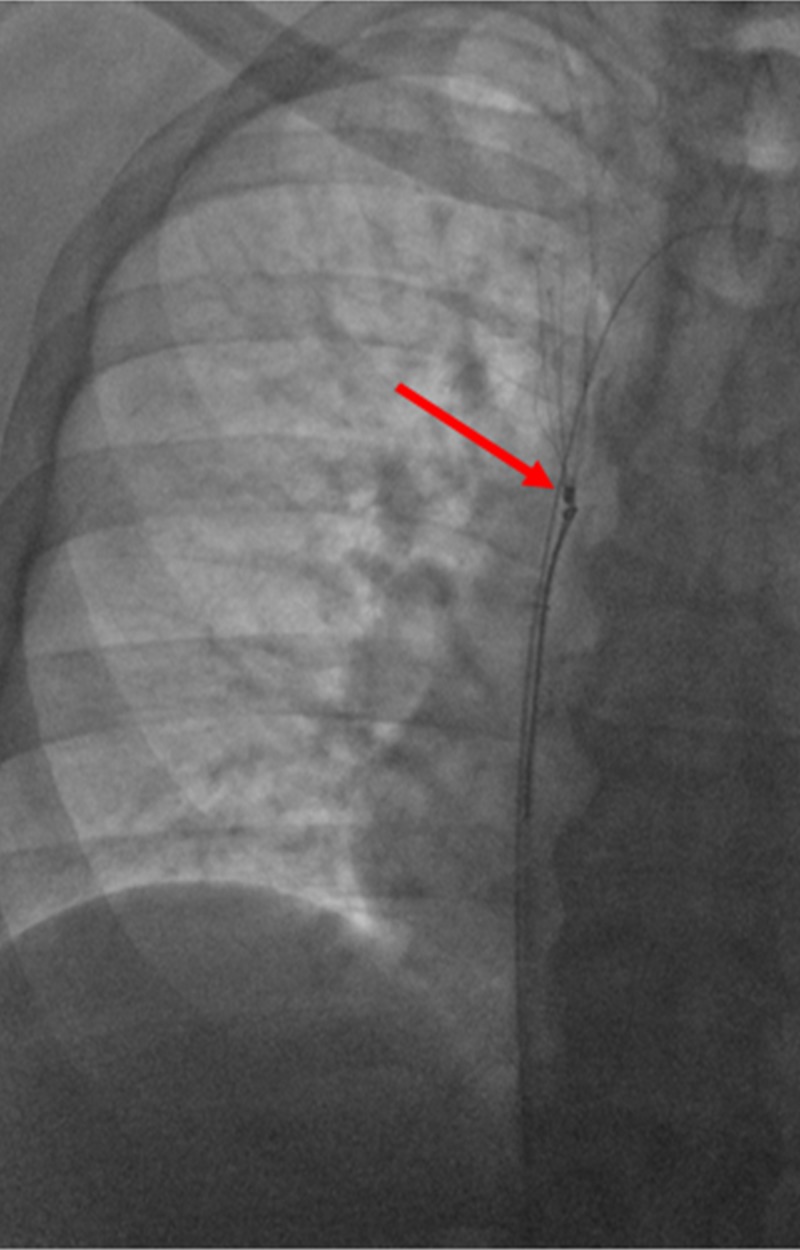
Snare Entrapment of the Superior Vena Cava Filter Engagement of the snare device is seen at the apex of the superior vena cava filter (red arrow), with a safety wire seen in the left subclavian vein.

Due to the medial angulation of the SVC filter tip, a super stiff Amplatz was used as a buddy wire and placed in the left brachiocephalic vein to guide the 8 French sheath medially. This provided support for the snare as it approached the filter tip (Figure 4).

**Figure 5:**
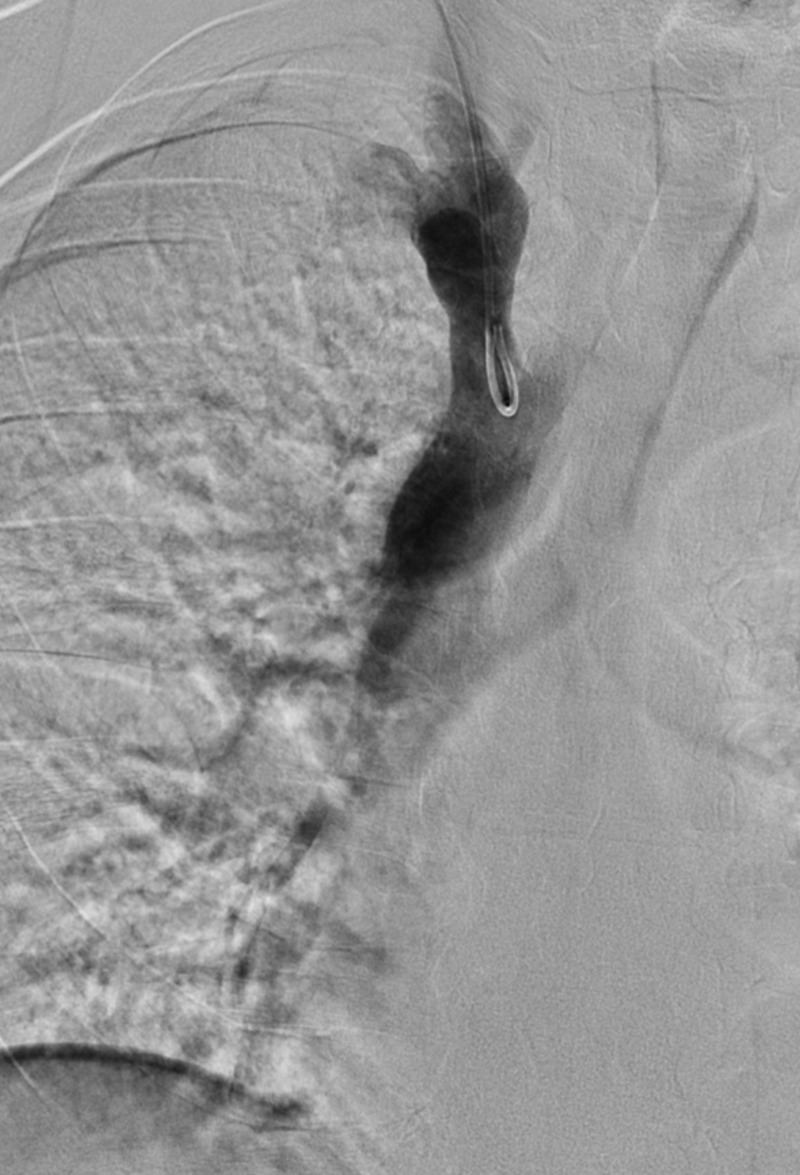
Post-interventional Venogram of the Superior Vena Cava A post-interventional venogram of the superior vena cava without evidence of injury or extravasation.

The filter was lassoed with a 10 mm Amplatz Gooseneck Snare (Medtronic, Minneapolis, MN) and removed without incident. A post-filter removal venography demonstrated an intact SVC without evidence of injury or extravasation (Figure 5).

The 8 French sheath was removed with the SVC filter entrapped within it.

Step 4: Retraction of the protective sheath and removal of the IVC filter

**Figure 6:**
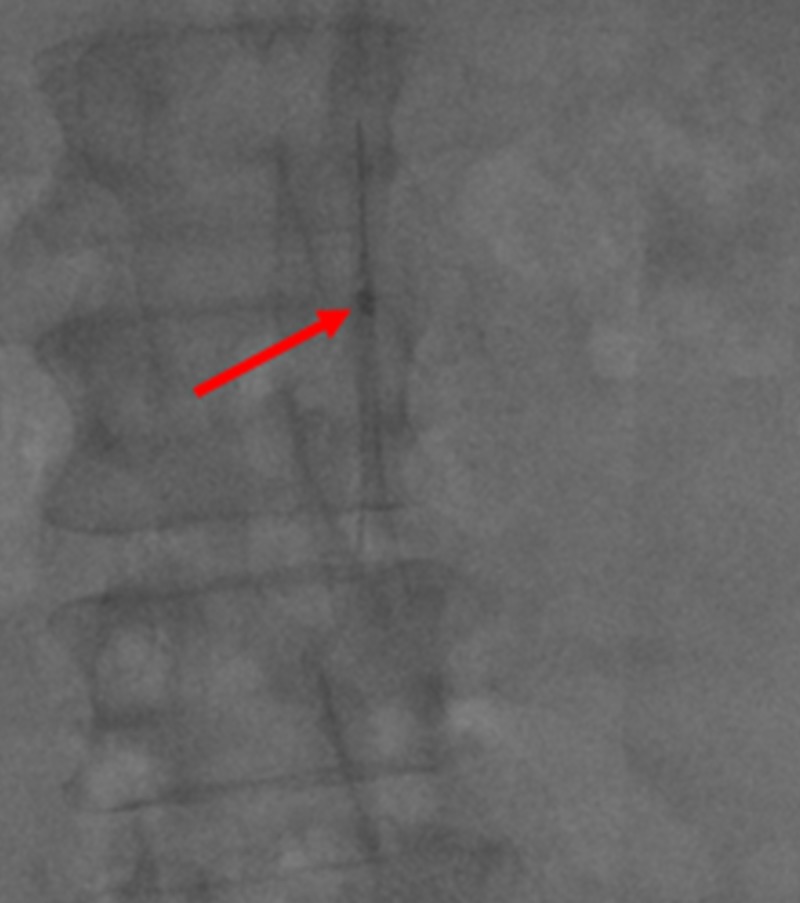
Retrieval of the Inferior Vena Cava Filter with a Snare Device The inferior vena cava filter was engaged and lassoed at the apex with a snare device (red arrow).

The introducer was placed back into the DrySeal Flex Sheath and carefully retracted back below the IVC filter. An 8 French Pinnacle Destination Guiding Sheath was placed over the right IJV guidewire to the tip of the IVC filter. The filter was lassoed with the 10 mm loop diameter Amplatz Gooseneck Snare and removed without incident (Figure 6).

**Figure 7:**
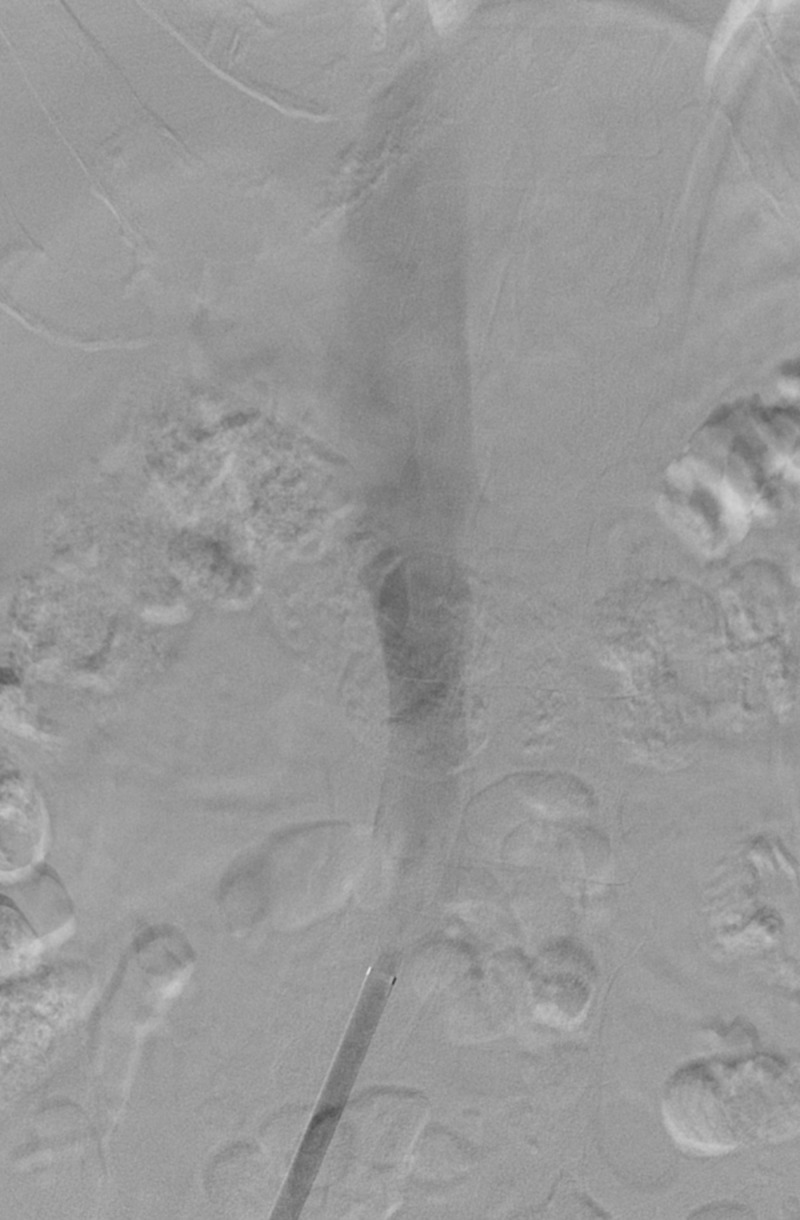
Post-interventional Venogram of the Inferior Vena Cava A post-interventional venogram of the inferior vena cava demonstrates no evidence of inferior vena cava injury.

A post-filter removal venography performed through the DrySeal Flex Sheath demonstrated an intact IVC without evidence of injury or extravasation (Figure 7).

All guidewires and catheters were then removed, and manual pressure was applied until hemostasis was obtained at both access sites.

## Discussion

The DrySeal Flex Sheath was chosen for its characteristic polytetrafluoroethylene (PTFE) coated outer surface, without texture or ribbing. Carefully placing this characteristically diminished friction coated sheath through the IVC filter would allow the inner sheath to move past the IVC filter without disturbance [6]. Additionally, having a larger 15 French diameter sheath would provide support and increase maneuverability of the inner sheath and wires. Any manipulation that needed to occur to advance wires or catheters when accessing the SVC was conducted entirely within the 15 French DrySeal Flex sheath, preventing any form of contact with the IVC filter at any point.

The SVC filter was specifically chosen to be removed first before the IVC filter to grant the operator a larger margin of error if needed. Though removal of SVC and IVC filters include the same risks and complications, such as disturbance, dislodgement, or fragmentation of the SVC filter, removing the IVC filter first would have been more consequential. SVC filter complication management has not been well documented and posed a higher risk for the operator had a complication arose [7]. Specifically, SVC filter fragmentation or movement would not allow for the operator to abort a maneuver with the time needed to correct errors made during the intervention. This is the result of anatomic location, where the SVC filter is much closer to the heart than the IVC filter. Conversely, IVC filter complication management is well documented and reported, allowing the operator to appropriately intervene if necessary [8]. The operator’s sequence of the presented intervention shown here is important. Performing the intervention in the reverse order would have defeated the purpose of the intervention, as the risks would have outweighed any benefit to the patient, especially if both filters could have been left in place initially.

This method for concurrent SVC and IVC filter removal decreased the possibility of filter migration or fragmentation. A large 15 French DrySeal Flex Sheath with a characteristic PTFE coated outer surface was a necessity in the success of this case. The protective outer sheath allowed for increased operator motility, ensuring minimal disturbance of the IVC filter while initially removing the superior filter in the SVC.

## Conclusions

Concurrent removal of SVC and IVC filters is a novel interventional method that has never been documented in medical literature before. The described sequential order of dual SVC/IVC filter removal is a well-thought-out progression of events that offers the best probability for successful retrieval of both filters in one session. Since the IVC filter apex is oriented toward the SVC filter and the SVC filter apex oriented toward the IVC filter, conventional removal of the filters is essentially impractical. Upon rare request and patient adequacy, using the intervention described offers the safest and most intuitive retrieval method of both filters.
